# Intra-abdominal desmoplastic small round cell tumour: staging and surveillance with ^18^F-FDG PET/CT following peritonectomy and HIPEC

**DOI:** 10.1259/bjrcr.20150434

**Published:** 2016-07-28

**Authors:** William Makis, Safwat Girgis

**Affiliations:** ^1^Department of Diagnostic Imaging, Cross Cancer Institute, Edmonton, Canada; ^2^Department of Pathology, University of Alberta Hospital, Edmonton, Canada

## Abstract

Intra-abdominal desmoplastic small round cell tumours are rare aggressive tumours of mesothelial origin with less than 60 cases reported in the literature. They are difficult to treat and the role of ^18^F-fludeoxyglucose positron emission tomography (PET)/CT scan in their management has not been established. A 41-year-old male presented with a 21-cm desmoplastic small round cell tumour and was managed with radiotherapy, surgery and chemotherapy, with each treatment monitored and guided by ^18^F-fludeoxyglucose PET/CT scan. We present the imaging findings of the serial PET/CT scans of this patient and their impact on management.

## Case report

**Figure 1.**
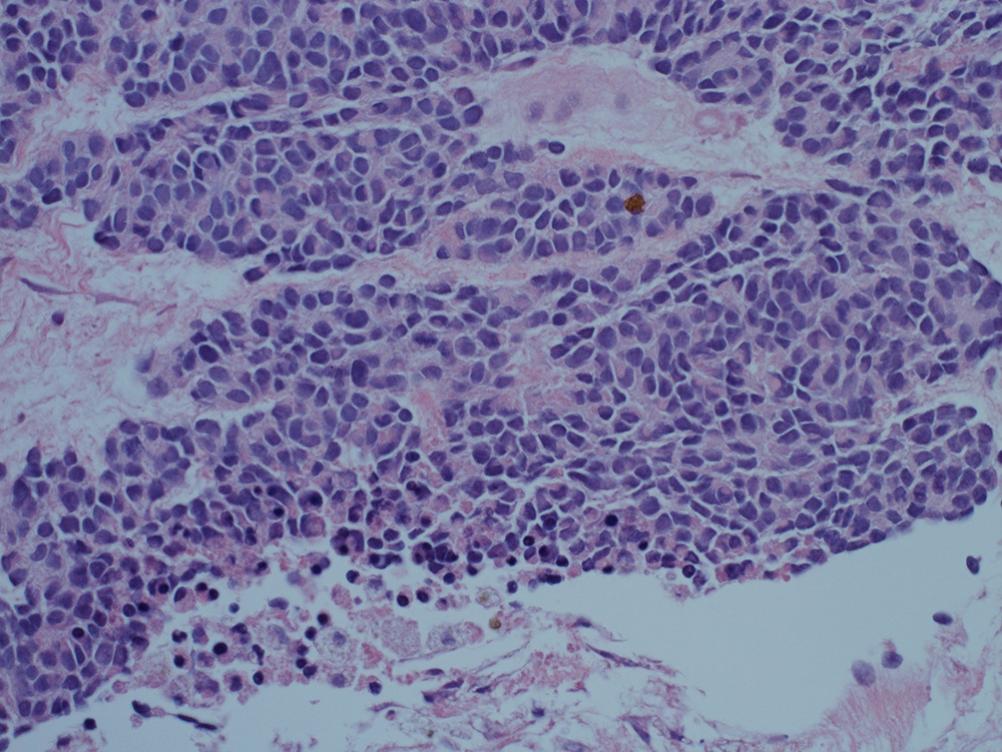
A high power 400× histological image of the routine preparation.

A 41-year-old male was found to have a large abdominal mass that was confirmed through biopsy to be a desmoplastic small round cell tumour (DSRCT). A high power 400× histological image of the routine preparation is shown (Figure 1). Microscopic examination showed the presence of epithelioid-like cells with a high nuclear–cytoplasmic ratio with moderate variability in size and shape. Cytologically, the tumour cells were intermediate in size with polarized, slightly scalloped nuclei and a small amount of eosinophilic cytoplasm. Infrequent mitotic figures were seen and geographic areas of necrosis were identified. Immunohistochemical staining revealed positivity to vimentin and staining with desmin showed a peculiar perinuclear dot pattern characteristic of DSRCT.^1^ CD56 was strongly positive, WT1 focally positive and other markers, including CD117, CD99, CD45, CD34, CD20, Cam 5.2, pancytokeratin, MYF4, S100, Actin, CD138, calretinin and synaptophysin, were negative. MIB-1 staining showed a high proliferative index with 60% positivity.

**Figure 2.**
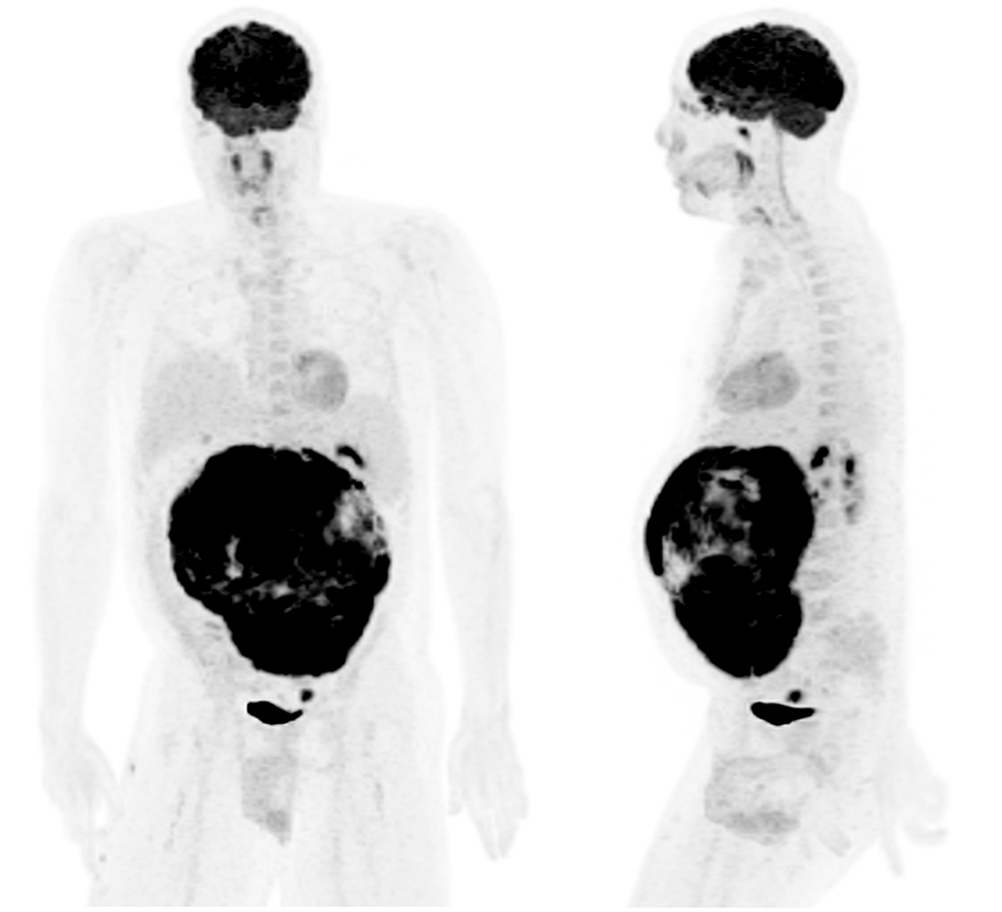
Staging ^18^F-fludeoxyglucose positron emission tomography/CT scan maximum intensity projection images showing a 25-cm abdominal mass and left pelvic peritoneal nodule.

**Figure 3.**
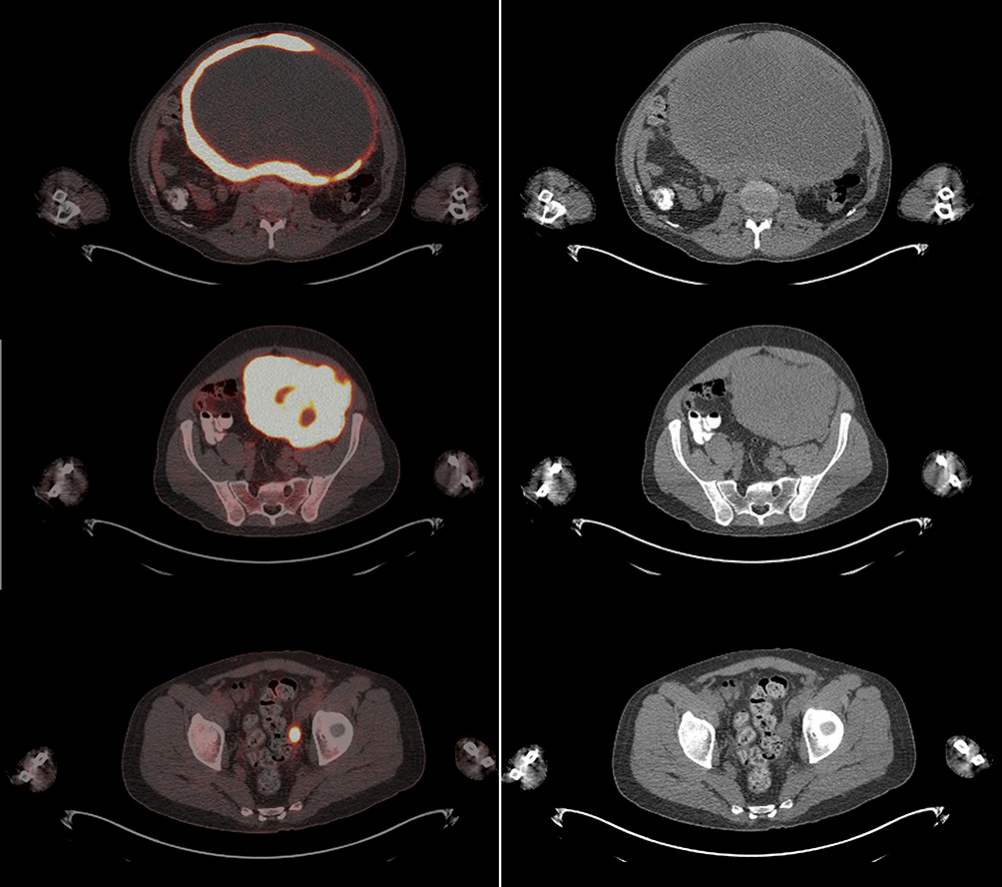
Staging positron emission tomography/CT fusion (left) and CT images (right) showing abdominal and peritoneal masses.

**Figure 4.**
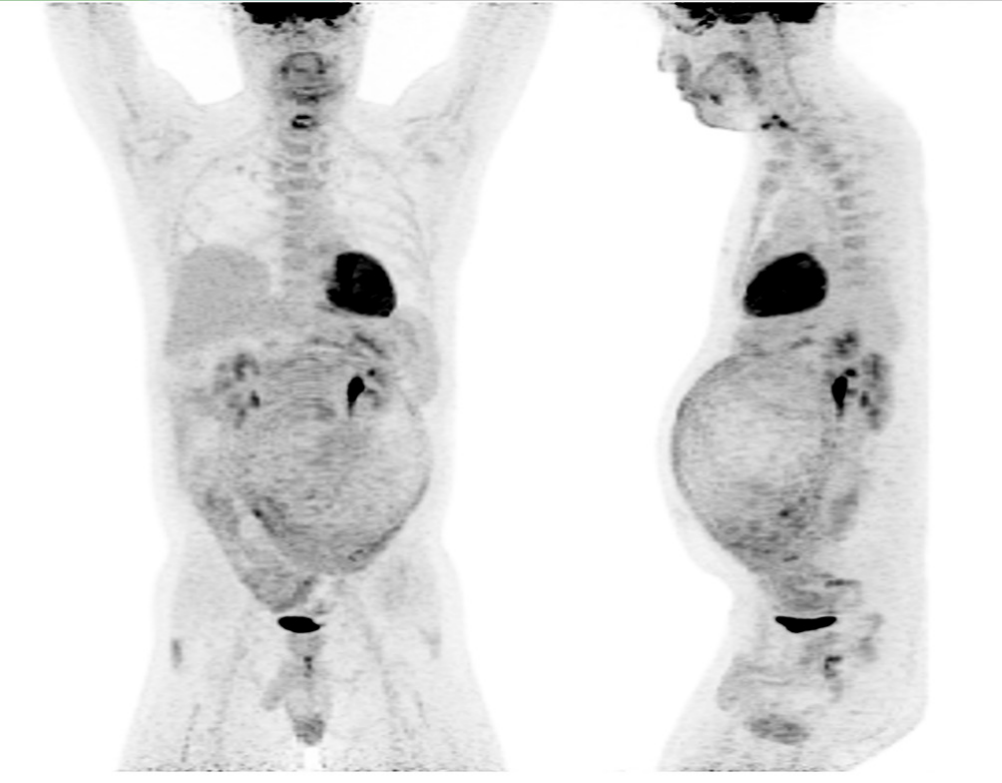
Post-radiation therapy positron emission tomography/CT scan (maximum intensity projection images) performed 4 weeks after the end of therapy showing excellent partial treatment response.

**Figure 5.**
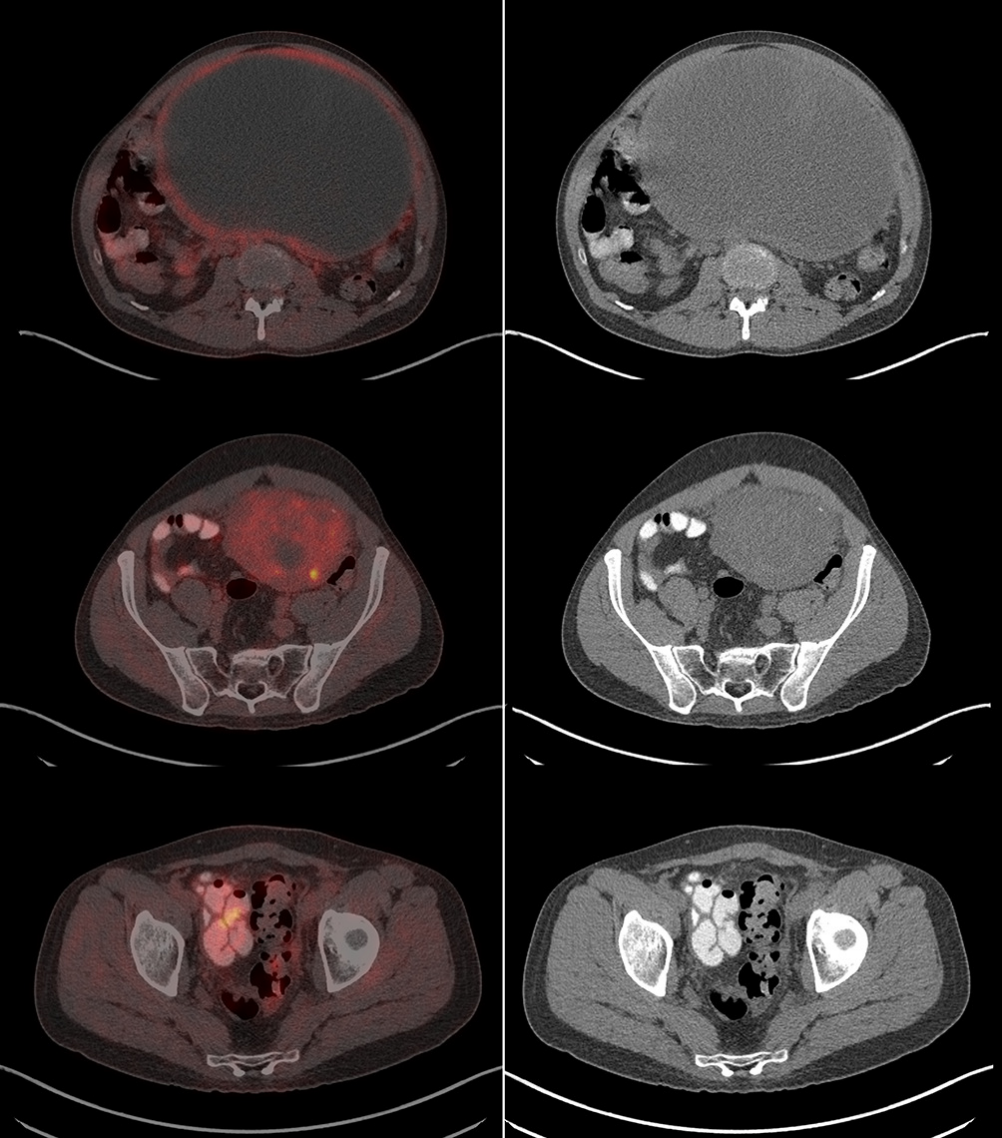
Post-radiation therapy positron emission tomography/CT fusion (left) and CT images (right) showing treatment response in the abdominal and peritoneal masses.

**Figure 6.**
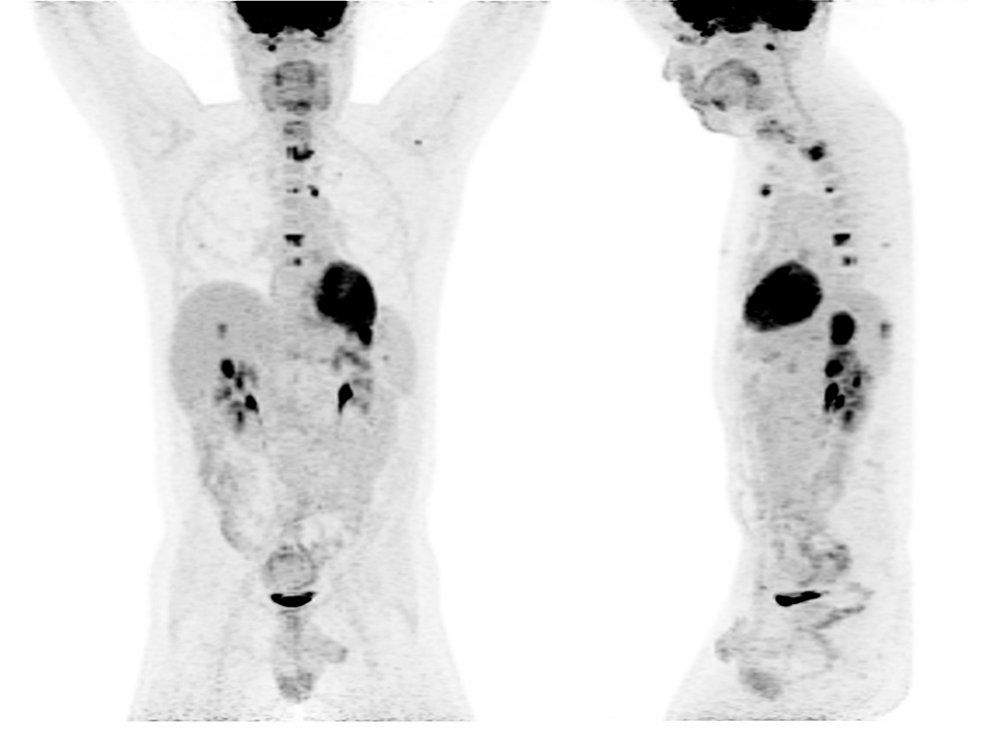
Post-surgery/chemotherapy positron emission tomography/CT scan performed 6 months after the surgery showing failure of therapy.

**Figure 7.**
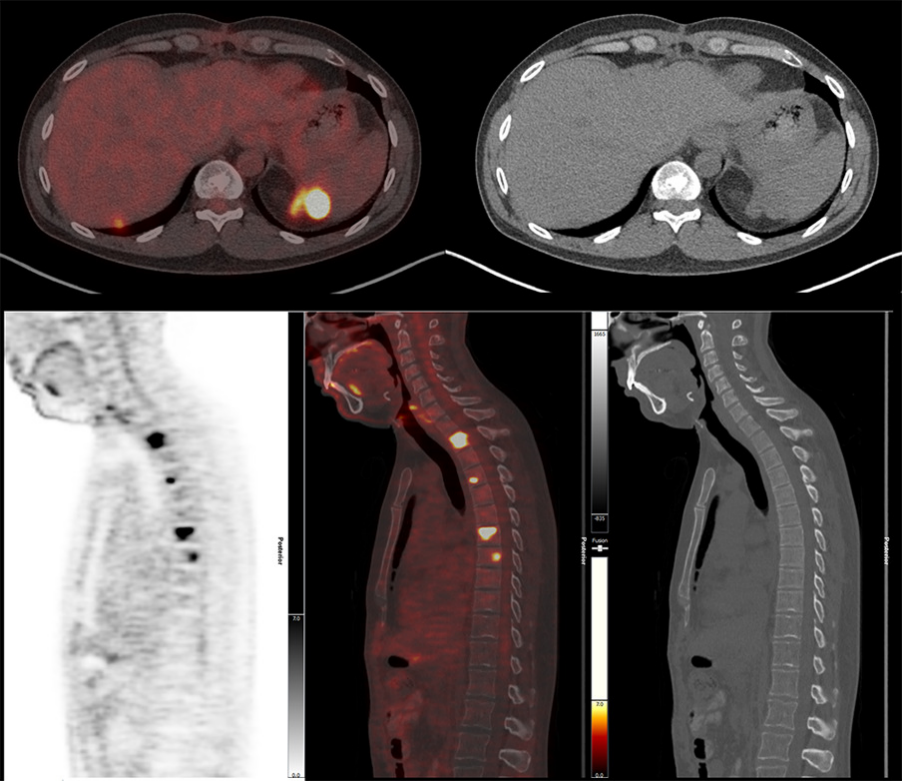
Post-surgery/chemotherapy positron emission tomography (bottom left), positron emission tomography/CT fusion (top left, bottom middle)/CT and CT images (top right, bottom right) showing new peritoneal and bone metastases.

**Figure 8.**
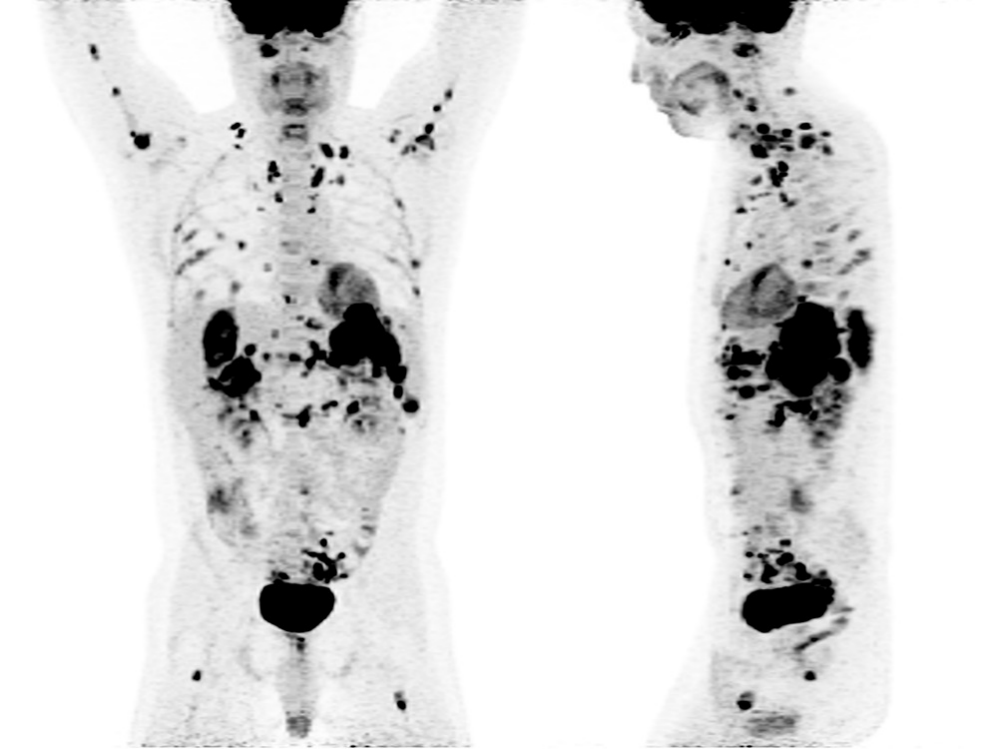
A follow-up positron emission tomography/CT scan (maximum intensity projection images) performed 3 months later showing extensive soft tissue and bone progression, at which point the patient refused further chemotherapy and passed away a few months later.

CT imaging showed an abdominal mass measuring 21 × 11 × 14 cm and the patient was treated with one cycle of chemotherapy with cyclophosphamide, adriamycin, vincristine, ifosfamide and etoposide; however, his mass progressed to 25 × 16 × 22 cm. ^18^F-fludeoxyglucose positron emission tomography (^18^F-FDG PET)/CT scan showed a mostly necrotic 25-cm abdominal mass with a maximum standardized uptake value (SUV_max_) of 18.5 with limited peritoneal disease (left pelvic nodule measuring 2.0 × 1.4 cm with SUV_max_ of 13.9) (Figures 2 and 3), which altered the treatment strategy from curative to palliative, and palliative radiation therapy (RT) was initiated. The patient received 5000 cGy to 90% of the planning target volume in 25 fractions over 6 weeks. Post RT ^18^F-FDG PET/CT scan performed 4 weeks after the end of therapy showed an excellent treatment response (abdominal mass SUV_max _of 18.5 decreased to 7.7, pelvic mass SUV_max _of 13.9 decreased to 2.4) (Figures 4 and 5). Based on the excellent response to therapy, as determined by the PET/CT scan, the patient opted for an aggressive approach with an attempt at curative resection of the tumour with peritonectomy and heated intraperitoneal chemotherapy (HIPEC), which was performed with cisplatin, 50 mg m^−2^ of body surface area (92.5 mg) in 4.5 l of 1.5% dianeal solution. The tumour was found to be arising from the transverse colon mesentery and was completely resected along with the left pelvic nodule. Unfortunately, a post-operative PET/CT scan performed 6 months after the surgery showed new peritoneal lesions (perisplenic soft tissue SUV_max_ of 18.2) and numerous bone metastases in the left humerus, cervical and thoracic spine, and ribcage (Figures 6 and 7). He had more RT from C7 to T8, and a follow-up PET/CT scan 3 months later showed extensive soft tissue progression (Figure 8). The patient refused further chemotherapy and passed away a few months later (20 months post-histological diagnosis, 12 months post-surgery/HIPEC).

## Discussion

DSRCTs are rare aggressive tumours of mesothelial origin, with less than 60 cases having been reported in the literature.^1,2^ They affect young males with a ratio of 5 : 1 and occur mainly in the abdomen and pelvis. They have a tendency to spread along the peritoneum and usually present at an advanced stage with a bulky primary mass, peritoneal seeding and distant metastases. Histological features include poorly differentiated small round cells within a desmoplastic stroma.^3,4^ Bulky, lobulated heterogeneous soft tissue peritoneal masses are a common CT feature.^5^ No consensus has yet been reached concerning the optimal strategy for managing DSRCT.^6^ ^18^F-FDG PET/CT scan is crucial in the staging of DSRCT,^2,4^ and several cases of serial PET/CT imaging have been described, although an optimal PET/CT imaging plan has not yet been determined.^6,7^ In one series of eight children (aged 2–20 years, median age 11 years), six patients with no abnormal ^18^F-FDG uptake on PET/CT scans performed after the end of therapy had excellent long-term outcomes (progression-free survivals of 2–10 years), while one patient who showed a partial response on PET/CT scan during treatment had a poor outcome, dying of disease 1.3 years from diagnosis.^8^ In our case, the staging PET/CT scan determined the extent of the disease, and a post-RT PET/CT scan showed excellent partial treatment response. These results were promising enough to allow the oncologists to offer the patient an aggressive treatment approach that has previously been described only in the paediatric population (surgical debulking plus HIPEC), while being aware that the odds of a curative response were very poor.^9^ The patient opted for the aggressive treatment approach; however, post-surgery/HIPEC, the follow-up PET/CT scan showed therapeutic failure after 6 months. Multimodality treatment of DSRCT involving surgical debulking, radiotherapy and chemotherapy has been shown to improve the 3-year survival from 27% to 55%,^10^ and serial PET/CT imaging will likely play an integral role in evaluating the response to each treatment modality and guiding the sequence and direction of the treatment plan.

## Learning points

1. DSRCTs are rare and aggressive tumours that are difficult to treat and the role of ^18^F-FDG PET/CT scanning has not been established.
2. Staging with ^18^F-FDG PET/CT can determine the full extent of the disease and direct initial management towards a curative or palliative approach.
3. A multimodality treatment approach has shown improvement in the overall survival, and serial ^18^F-FDG PET/CT imaging performed after each treatment modality will likely be vital in assessing the response to therapy and directing the next steps in management.

## Consent

Informed consent to publish this case (including images and data) was obtained and is held on record.
